# Transcriptome profiling identifies regulators of pathogenesis in collagen VI related muscular dystrophy

**DOI:** 10.1371/journal.pone.0189664

**Published:** 2017-12-15

**Authors:** Russell J. Butterfield, Diane M. Dunn, Ying Hu, Kory Johnson, Carsten G. Bönnemann, Robert B. Weiss

**Affiliations:** 1 University of Utah, Departments of Pediatrics and Neurology, Salt Lake City, Utah, United States of America; 2 University of Utah, Department of Human Genetics, Salt Lake City, Utah, United States of America; 3 Neurogenetics Branch, National Institute of Neurological Disorders and Stroke, NIH, Bethesda, Maryland, United States of America; 4 Bioinformatics section, National Institute of Neurological Disorders and Stroke, NIH, Bethesda, Maryland, United States of America; Ecole normale superieure de Lyon, FRANCE

## Abstract

**Objectives:**

The collagen VI related muscular dystrophies (COL6-RD), Ullrich congenital muscular dystrophy (UCMD) and Bethlem myopathy (BM) are among the most common congenital muscular dystrophies and are characterized by distal joint laxity and a combination of distal and proximal joint contractures. Inheritance can be dominant negative (DN) or recessive depending on the type and location of the mutation. DN mutations allow incorporation of abnormal chains into secreted tetramers and are the most commonly identified mutation type in COL6-RD. Null alleles (nonsense, frameshift, and large deletions) do not allow incorporation of abnormal chains and act recessively. To better define the pathways disrupted by mutations in collagen VI, we have used a transcriptional profiling approach with RNA-Seq to identify differentially expressed genes in COL6-RD individuals from controls.

**Methods:**

RNA-Seq allows precise detection of all expressed transcripts in a sample and provides a tool for quantification of expression data on a genomic scale. We have used RNA-Seq to identify differentially expressed genes in cultured dermal fibroblasts from 13 COL6-RD individuals (8 dominant negative and 5 null) and 6 controls. To better assess the transcriptional changes induced by abnormal collagen VI in the extracellular matrix (ECM); we compared transcriptional profiles from subjects with DN mutations and subjects with null mutations to transcriptional profiles from controls.

**Results:**

Differentially expressed transcripts between COL6-RD and control fibroblasts include upregulation of ECM components and downregulation of factors controlling matrix remodeling and repair. DN and null samples are differentiated by downregulation of genes involved with DNA replication and repair in null samples.

**Conclusions:**

Differentially expressed genes identified here may help identify new targets for development of therapies and biomarkers to assess the efficacy of treatments.

## Introduction

The collagen VI related muscular dystrophies (COL6-RD), Ullrich congenital muscular dystrophy (UCMD) and Bethlem myopathy (BM) are inherited disorders of collagen VI characterized by distal joint laxity and a combination of distal and proximal joint contractures [1, 2]. COL6-RD are increasingly recognized disorders accounting for up to 30% of patients with congenital muscular dystrophy (CMD) phenotypes.[2, 3] COL6-RD are unusual among the muscular dystrophies in that both muscle and connective tissue are significantly affected.[4] Patients have muscle weakness and atrophy attributable to a defect in the muscle, but also have severe joint contractures, joint laxity, keratosis pilaris, and a predilection to keloid formation attributable to a connective tissue defect.[4] Children with COL6-RD have life-long neurologic, orthopedic, and pulmonary complications necessitating complex, multispecialty care.[4] Despite increased recognition of these disorders, there are no treatments.

In most CMD phenotypes the molecular defect results in a direct disruption of the stability sarcolemmal membrane and its linkages between the cytoskeleton and extracellular matrix (ECM). In COL6-RD, the pathogenic mechanisms are less well understood. While components of the ECM in muscle, including collagens I, III, and V, are produced by both the myofiber and muscle connective tissue (MCT) fibroblasts, collagen VI in the muscle is produced primarily by MCT fibroblasts.[5, 6] It is not clear how a defect in collagen VI results in a defect in the muscle.[4, 7] COL6-RD may constitute a “non-cell autonomous” disorder whereby the cell causing disease (MCT fibroblast) is different from the cell where disease is expressed (myofiber).[6] The mechanisms whereby a disruption of collagen VI in the ECM of muscle results in pathologic phenotype on the myofiber are not yet understood.[4, 7]

To better define the pathways disrupted by mutations in COL6-RD, we have used a transcriptional profiling approach with RNA-Seq to identify differentially expressed genes in cultured dermal fibroblasts from COL6-RD subjects and controls. Computational analysis of read count data from RNA-Seq experiments provide estimations of transcript isoforms and abundance on a genomic scale.[8, 9] To better assess the transcriptional changes induced by the presence abnormal collagen VI in the extracellular matrix (ECM) (as compared to its absence from the ECM); we have compared transcriptional profiles from subjects with dominant negative mutations and subjects with null mutations to transcriptional profiles from controls.

## Materials and methods

### Subjects/Samples

Thirteen COL6-RD subjects and 6 control subjects were included in the study. (Table 1) All patients included in the study have clinical features typical of UCMD including early onset hypotonia with proximal contractures, distal hyperlaxity, and hyperkeratosis pilaris with loss of ambulation or trajectory toward loss of ambulation by teenage years. Dominant negative (DN) mutations including glycine substitutions and exon skipping mutations in the triple helical (TH) region of any of the collagen VI genes are the most common mutational mechanism in COL6-RD. DN mutations allow residual, abnormal collagen VI in the ECM, while null mutations produce no collagen VI in the ECM. We selected eight subjects with dominant negative mutations, 4 with the glycine substitution p.Gly284Arg or p.G281Arg in the TH domain in *COL6A1* and 4 with exon skipping mutations involving exon 16 of the *COL6A3* gene, thus representing the most common DN mutations. Five subjects with COL6-RD due to recessive, null mutations were also included. This study was approved by the Institutional Review Board of the National Institute of Neurological Disorders and Stroke, National Institutes of Health and the University of Utah (IRB registrations: 30923, Utah; 12N0095, NIH). Written informed consent and appropriate assent were obtained from all participating subjects.

**Table 1:** Mutations in COL6-RD subjects with RNA-seq.

Subject	Age at biopsy (yrs)	Mutation type	Gender	Gene	Exon	Mutation
**2**	3	DN, exon 16 skipping	F	*COL6A3*	16	c.6210 +1G>A, p.Gly2053_Pro2070del
**3**	3	DN, exon 16 skipping	M	*COL6A3*	16	c.6210 +1G>A, p.Gly2053_Pro2070del
**4**	11	DN, exon 16 skipping	F	*COL6A3*	16	c.6210 +1G>A, p.Gly2053_Pro2070del
**5**	22	DN, exon 16 skipping	F	*COL6A3*	16	c.6210 +1G>A, p.Gly2053_Pro2070del
**6**	15	DN, glycine substitution	M	*COL6A1*	9	c.841G>A, p.Gly281Arg
**7**	10	DN, glycine substitution	F	*COL6A1*	9	c.850G>A, p.Gly284Arg
**18**	14	DN, glycine substitution	F	*COL6A1*	9	c.850G>A, p.Gly284Arg
**19**	12	DN, glycine substitution	M	*COL6A1*	9	c.850G>A, p.Gly284Arg
**8**	0.5	Null	M	*COL6A2*	2 17	c.115+2T>C ^1^ c.1402C>T, p.Arg468*
**9**	9	Null	M	*COL6A1*	21	c.1451del, p.P484Qfs*21 (homozygous)
**10**^2^	4	Null	M	*COL6A2*	all	47Kb genomic deletion including *COL6A2* 1.61Mb genomic deletion including *COL6A1*, and *COL6A2*
**11**	3	Null	F	*COL6A2*	26 7	c.2386A>T, p.K796* c.856-3C>G^3^
**15**	9	Null	M	*COL6A3*	2	c.53C>A; p.S18* (homozygous)

^1^Disruption of splicing donor results in activation of cryptic splice site and 9bp insertion to cDNA. This allele is inherited from unaffected father. Unaffected mother carries the nonsense allele.

^2^P2 from Foley et al, *Ann Neurol* 2011.[10] The 1.61 Mb deletion includes 17 genes: *ADARB1*, *POFUT2*, *COL18A1*, *SLC19A1*, *PCBP3 COL6A1*, *COL6A2*, *FTCD C21orf56*, *LSS MCM3AP*, *C21orf57*, *C21orf58*, *PCNT*, *DIP2A*, *S100B*, and *PRMT2*. Unaffected mother and brother both carry this allele.

^3^Splicing variant resulting in creation of novel splice acceptor (a**c**ag|GG>a**g**ag|GG.). Result is 2 basepair insertion to cDNA and out-of-frame transcript.

Fibroblasts derived from skin biopsy samples were grown in Dulbecco’s modified Eagle medium with 10% FBS and 1% Penicillin/Streptomycin in a 6-well plate in 5% CO2 at 37°C until 80% confluence. Cells were continuously cultured in the presence of 25ug/ml L-ascorbic acid phosphate (Wako, Osaka, Japan) for 72 hours and then changed to medium without L-ascorbic acid phosphate for 16 hours before the RNA extraction using RNeasy Mini Kit (QIAGEN, cat# 74106).

### Sequencing

Total RNA was obtained from previously existing dermal fibroblast cell lines from COL6-RD individuals with dominant negative or null mutations and from existing control fibroblast cell lines. Poly-A purified sequencing libraries were produced from total RNA using the Illumina TruSeq RNA Sample Prep Kit (Illuminia, Inc., San Diego, CA) according to the manufacturer’s protocol. Sequencing was performed using the Illumina HiSeq 2000 instrument with a 50 cycle single-end protocol at the University of Utah Microarray and Genomic Analysis Core. Sequenced reads were processed for quality using FASTX toolkit version 0.0.13 (http://hannonlab.cshl.edu/fastx_toolkit/) to clip adapter sequences and filter for minimum quality score of 15 or greater in 80% of bases per read.

### Differential expression analysis

Read mapping and differential expression analysis were performed using the TopHat-Cufflinks pipeline using a reference transcriptome based on (GRCv37 / UCSC hg19) reference genome.[11] Alignment of filtered/clipped reads to the reference sequence was performed using Tophat2 (v2.0.10) with default parameters.[8] Transcript abundances were estimated and normalized for transcript length and for read depth and reported as fragments per kb (in the transcript model) per million reads mapped (FPKM) using Cufflinks (v2.2.1).[9] Differential expression was determined using Cuffdiff2 with pairwise comparisons for 1) control vs. dominant negative mutants, 2) control vs. null mutants, and 3) dominant negative mutants vs. null mutants.[12] Our analysis used the –G option to restrict the analysis to the reference genome, -u to correct for multi-reads, and–b option to implement the fragment bias correction algorithm.[13] Genes were considered differentially expressed and included in ontology and pathway analysis when the FDR-adjusted p-value was <0.05. We identified enriched gene ontology categories and KEGG pathways among differentially expressed genes using GOSeq, an algorithm specifically designed for RNA-Seq data.[14] Gene ontology terms were considered significantly enriched if the FDR adjusted p-value was <0.01. Redundant gene ontology categories were parsed using Revigo, an online tool to identify redundant GO terms.[15] GO terms with frequency > 1%, dispensability >0.2, and uniqueness <0.5 were filtered from the list of enriched terms. The data, including raw sequence files for each subject have been deposited in NCBI’s Gene Expression Omnibus and are accessible through GEO Series accession number GSE103270 (https://www.ncbi.nlm.nih.gov/geo/query/acc.cgi?acc=GSE103270).

## Results

### Mapping and differential expression analysis

RNA sequencing (RNA-Seq) was completed for 13 COL6-RD samples and 6 control samples. Average sequencing depth was 32.8 million reads per sample (range 24.5–47.6 million). Filtering adaptor sequences and poor quality reads removed a mean 7.5% reads per sample. Mapping using TopHat was successful for a mean of 90.7% of total reads. There were no significant differences in sequencing depth or mapping efficiency between samples by mutation group (control vs. DN vs. null) or by gender (M vs. F).

Expression values were normalized to library size and transcript length using Cufflinks and reported as fragments per kilobase of transcript per million reads mapped (FPKM). As expected, the most highly expressed genes were ECM components including collagen I, fibronectin and vimentin. The three genes forming collagen VI (*COL6A1*, *COL6A2*, and *COL6A3*) were all in the top 100 highly expressed genes. Expression of *COL6A3* was about 1/3 the level of expression of *COL6A1* and *COL6A2*. As expected, collagen VI null samples had decreased expression for their null alleles compared to controls, while the expression of the non-mutated collagen VI genes does not appear to be increased in compensation for absence of the null allele (Table 2). Expression was not completely ablated by the null allele in subject 15 with an expression of *COL6A3* of 26% of the control level. Residual expression of the mutant *COL6A3* transcript in this sample suggests that some mutant transcripts may escape nonsense-mediated decay. Absence of collagen VI in cultured fibroblasts from this sample suggests that these transcripts do not produce significant amounts of collagen VI (S1 Fig). Expression of the *COL6A4P1*, *COL6A4P2*, *COL6A5* and *COL6A6* genes was below detection levels. Multidimensional scaling analysis based on estimated expression levels demonstrates that dominant negative samples are distinct from null and control samples (S2 Fig). Clustering of control and null samples is less distinct, but suggestive that groups are different from each other.

**Table 2:** Expression levels for *COL6A1*, *COL6A2*, and *COL6A3* genes.

Gene	Reference ID	Length (bp)	Mean FPKM by Mutation Class			Individual FPKM for null subjects and % of control^1^				
			Control (n = 6)	Dominant Negative (n = 8)	Null (n = 5)	Subject 8	Subject 9	Subject 10	Subject 11	Subject 15
*COL6A1*	NM_001848	4225	1981.9	2199.0	1195.3	938.4	**56.2**	1124.8	2296.3	1560.8
						(47%)	**(3%)**	(57%)	(116%)	(79%)
*COL6A2*	NM_001849	3439	2371.3	2732.4	793.8	**139.2**	1730.4	**1.4**	**226.4**	1871.6
						**(6%)**	(73%)	**(0%)**	**(10%)**	(79%)
*COL6A3*	NM_004369	10,581	690.3	813.4	712.6	541.7	1089.5	791.8	959.5	**180.6**
						(78%)	(158%)	(115%)	(139%)	**(26%)**

^1^FPKM and percent of control mean FPKM for individual samples with null mutations. Bold figure represents a null mutation in that gene and demonstrates marked decrease in transcription level.

**Fig 1:**
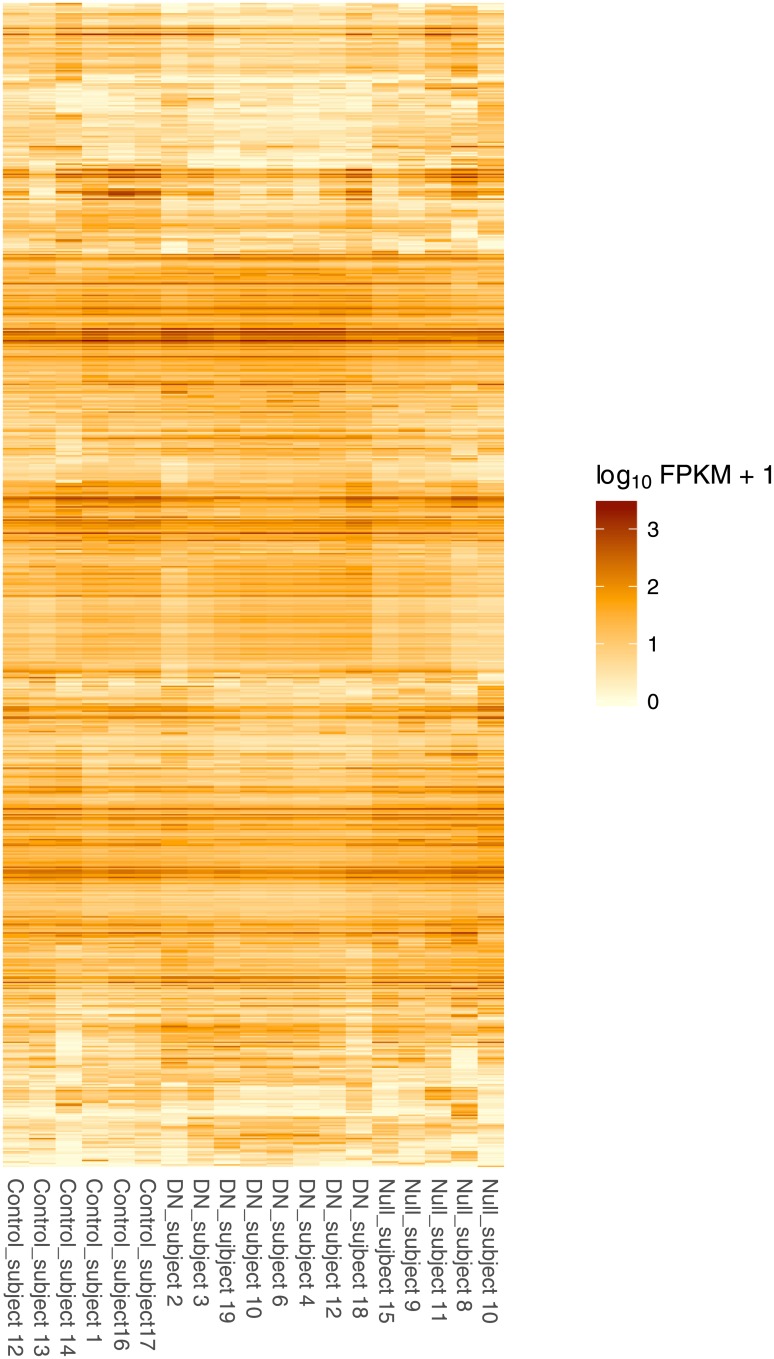
Heatmap of FPKM expression values for 1246 differentially expressed genes in cultured fibroblasts from COL6-RD subjects and controls. Heatmap generated using the *csHeatmap* function in cummeRbund[11] and including a non-overlapping list of DE genes in DN vs control, Null vs control, and DN vs Null comparisons.

Differential expression analysis was performed using Cufflinks2.[12] We identified 586 differentially expressed genes (FDR-adjusted p-value <0.05) in comparison of DN to Control samples, and 341 differentially expressed genes in Null vs. Control samples. Comparison of DN to Null samples revealed 868 differentially expressed (DE) genes. When considering all affected samples together, (DN and null compared to control) 171 genes were differentially expressed. Taken together we identified 1246 genes differentially expressed in one or more comparisons (Fig 1). A complete list of differentially expresses genes for each comparison (DN vs. control, Null vs. control, DN vs. Null, and any mutation vs. control) is included in electronic format including test statistics and an estimation of fold change (S1 File).

### Gene ontology and pathway analysis

We identified 329 enriched GO terms in the DN vs. control group, 311 in the Null vs. control group, and 196 in the DN vs. null group. After parsing GO terms for redundancy, 76 GO terms remained in the DN vs. control group, 61 in the Null vs. control group, and 53 in the DN vs. Null group. The top enriched non-redundant GO terms in dominant negative vs. control fibroblasts came from categories centered on three primary themes, extracellular matrix, adhesion, and immune response (Table 3, S2 File). A similar pattern of enriched GO terms was seen in enrichment of DE genes in control vs. null fibroblasts. In contrast, enriched GO ontologies in DN vs. Null fibroblasts centered on DNA replication/repair and proliferation.

**Table 3:** Enriched gene ontology terms among differentially expressed genes in COL6-RD subjects with dominant negative or null mutations compared to control^1^.

Dominant Negative vs. Control		Null vs. Control		Dominant Negative vs. Null	
Term ID	FDR	Term ID	FDR	Term ID	FDR
GO:0005615; extracellular space	1.6E-17	GO:0044707; single-multicellular organism process	5.1E-16	**GO:0006271; DNA strand elongation involved in DNA replication**	**8.4E-16**
GO:0032501; multicellular organismal process	3.3E-14	GO:0032501; multicellular organismal process	5.5E-16	GO:0032501; multicellular organismal process	1.9E-13
GO:0044707; single-multicellular organism process	7.9E-14	GO:0031012; extracellular matrix	1.5E-13	GO:0044707; single-multicellular organism process	9.0E-11
GO:0031012; extracellular matrix	2.6E-13	GO:0005578; proteinaceous extracellular matrix	3.7E-12	GO:0005615; extracellular space	3.8E-10
GO:0007155; cell adhesion	3.3E-13	GO:0007155; cell adhesion	1.4E-09	**GO:0042555; MCM complex**	**2.4E-08**
GO:0042127; regulation of cell proliferation	1.0E-10	**GO:0033993; response to lipid**	**2.4E-08**	GO:0008283; cell proliferation	3.0E-07
GO:0008283; cell proliferation	1.6E-10	GO:0008283; cell proliferation	7.4E-08	GO:0007155; cell adhesion	3.7E-07
**GO:0005539; glycosaminoglycan binding**	**6.7E-09**	GO:0030198; extracellular matrix organization	2.8E-07	**GO:0007267; cell-cell signaling**	**8.3E-07**
**GO:0002376; immune system process**	**3.4E-08**	GO:0042127; regulation of cell proliferation	1.4E-06	GO:0042127; regulation of cell proliferation	8.2E-06
GO:0006928; cellular component movement	7.0E-08	GO:0005201; extracellular matrix structural constituent	4.2E-06	GO:0031012; extracellular matrix	1.2E-05
**GO:0005125; cytokine activity**	**9.4E-08**	**GO:0016477; cell migration**	**5.0E-05**	**GO:0005657; replication fork**	**1.7E-05**
GO:0030198; extracellular matrix organization	1.3E-06	**GO:0005102; receptor binding**	**8.1E-05**	GO:0022414; reproductive process	2.6E-05
GO:0008201; heparin binding	2.3E-06	**GO:0001775; cell activation**	**1.5E-04**	GO:0044702; single organism reproductive process	1.4E-04
GO:0004888; transmembrane signaling receptor activity	6.6E-06	GO:0022414; reproductive process	6.8E-04	**GO:0016477; cell migration**	**2.5E-04**
GO:1901681; sulfur compound binding	9.0E-06	**GO:0031225; anchored component of membrane**	**6.9E-04**	**GO:0006928; cellular component movement**	**6.6E-04**

^1^Bold terms represent enriched ontologies unique to the differentially expressed genes within a comparison group; the top 15 significant, non-redundant categories are represented here (ranked by FDR). Total significant GO terms for Dominant Negative vs. Control is 528, for Null vs Control is 505, and for Dominant Negative vs. Null is 379. Significant DE genes present in each enriched GO category are specified in the supplemental table (S2 File).

**Fig 2:**
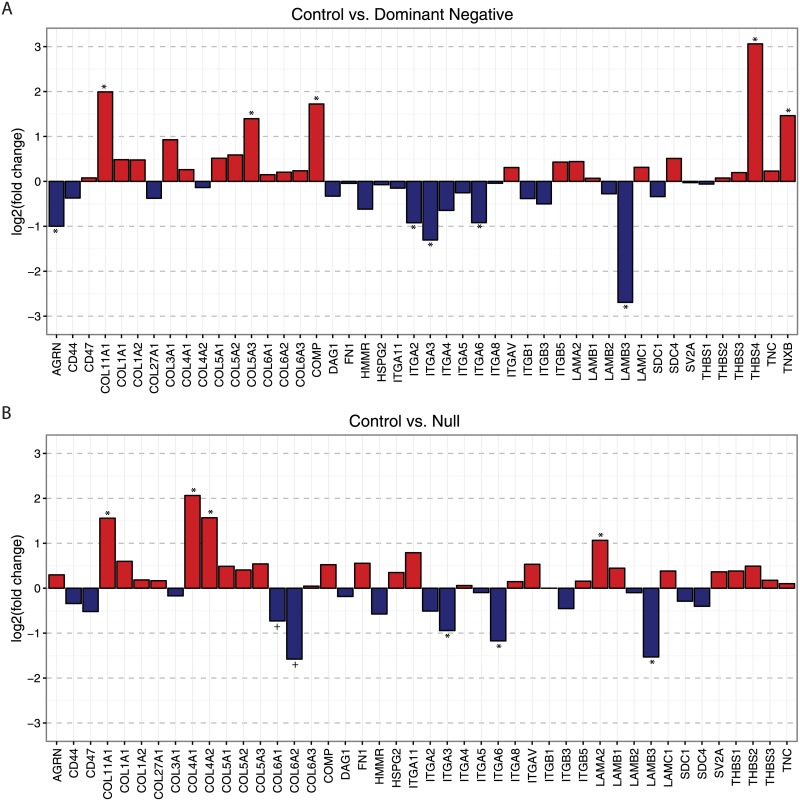
Differential expression of genes in the ECM-receptor interaction pathway. Differential expression of genes in the ECM-receptor interaction pathway (hsa04512) for control vs. dominant negative mutation (upper panel) and control vs null mutation (lower panel). Log_2_ fold change is plotted for each gene in the pathway. Genes with significant differential expression (FDR-adjusted p-value was <0.05) are marked with an *. *COL6A1* and *COL6A2* (marked with + in the lower panels) are decreased in null samples as expected due to their null allele.

Enrichment of KEGG pathways reflected similar themes with significant enrichment of the ECM-receptor interaction pathway (hsa04512) and the cytokine-cytokine receptor interaction pathway (hsa04060) in the DN vs. control and DN vs Null groups (S1 Table). Structural ECM components such as collagens were increased generally, with significant upregulation of *COL11A1* and *COL5A3* in DN vs. control fibroblasts and *COL11A1*, *COL4A1* and *COL4A2* in null vs. control samples (Fig 2). While not a part of the hsa04512 pathway other collagen genes were also significantly upregulated: *COL14A1*, *COL15A1*, *COL7A1*, and *COL8A2* in DN vs. control fibroblasts and *COL14A1*, *COL15A1*, *COL8A2* in null vs. control fibroblasts. In contrast, integrins and other signaling/adhesion components of the matrix were downregulated, including: *ITGA2*, *ITGA3*, *ITGA6* and *LAMB3* in DN vs. control and *ITGA3*, *ITGA6*, and *LAMB3* in null vs. control.

**Fig 3:**
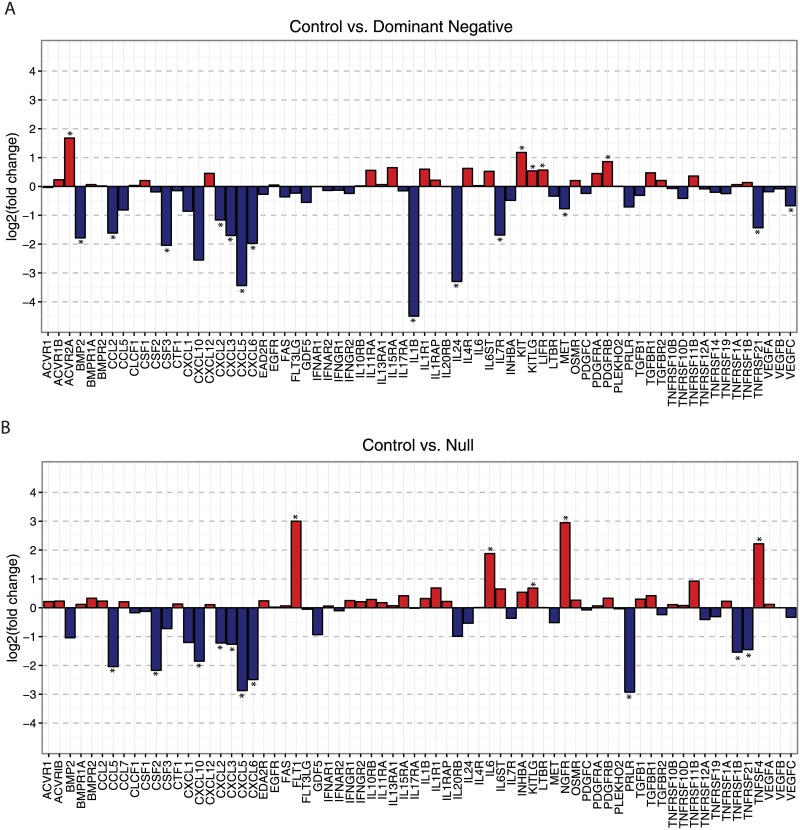
Differential expression of genes in the cytokine-cytokine receptor interaction pathway. Differential expression of genes in the cytokine-cytokine receptor interaction pathway (hsa04060) for control vs. dominant negative mutation (upper panel) and control vs. null mutation (lower panel). Log_2_ fold change is plotted for each gene in the pathway. Genes with significant differential expression (FDR-adjusted p-value was <0.05) are marked with an *.

DE genes in the cytokine-cytokine interaction pathway (hsa04060) include downregulation of a cluster of ELR (glutamic acid-leucine-arginine)-positive CXC chemokines, including: *CXCL2*, *CXCL3*, *CXCL5*, and *CXCL6* in both the DN vs. control and the Null vs. control groups **(**Fig 3). *IL8*, another ELR-positive CXC chemokine is also significantly downregulated. Other notable DE genes in this pathway include upregulation of *ACVR2A* and down regulation of *IL1B* and *IL24* in the DN vs. control group and upregulation of *FLT1*, *IL6*, *NGFR*, and *TNFSF4* in the Null vs. control group. *PRLR* is significantly downregulated in the Null vs. control group, but not differentially expressed in the DN vs. control group.

**Fig 4:**
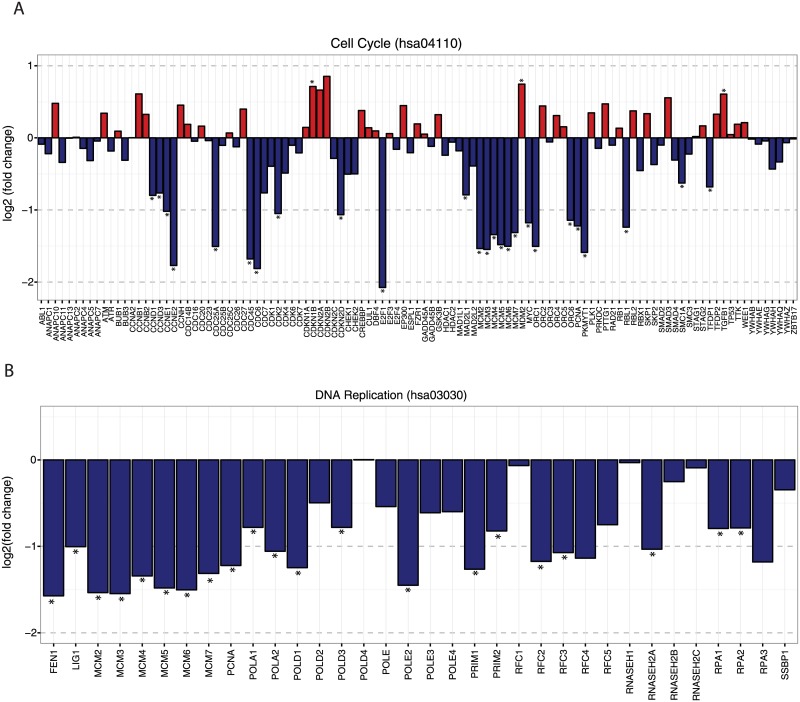
Differential expression of genes in the cell cycle pathway. Differential expression of genes in the cell cycle pathway (hsa04110, upper panel) and DNA replication (hsa03030, lower panel) for COL6-RD dominant negative vs. null (lower panel). Log_2_ fold change is plotted for each gene in the pathway (positive values reflect increased expression in the dominant negative mutants). Genes with significant differential expression (FDR-adjusted p-value was <0.05) are marked with an *.

Enrichment of KEGG pathways in comparing the DN vs. null group cluster strongly around a single theme: proliferation and cell division. Enriched pathways include both cell cycle (hsa04110) and DNA replication (hsa03030) pathways (Fig 4). In both pathways, null samples show downregulation of almost all genes compared to DN including clusters of cyclin genes, polymerases, and minichromosome maintenance complex (MMC) genes. *CDKN1B* and *TGFB1* were upregulated in the null compared to DN; however, consistent with the effects from other genes in the pathway, both *TGFB1* and *CDKN1B* are negative regulators of proliferation. Mismatch repair (hsa03430), pyrimidine metabolism (hsa00240), and nucleotide excision repair (hsa03420) pathways all showed similar findings (S1 Table).

## Discussion

COL6-RD are the paradigmatic disorders of the extracellular matrix of muscle and are unique among the CMD in that both muscle and connective tissue are affected. Since collagen VI is produced by resident interstitial fibroblasts, the downstream pathological effects on muscle cells are cell non-autonomous.[6] Thus the effect of mutant collagen VI on fibroblasts is critical to understanding the mechanisms whereby a disruption of collagen VI in the muscle ECM confers pathologic consequences on the myofiber. To approach this question, we used a transcriptome profiling approach with RNA-Seq in cultured dermal fibroblasts from individuals with COL6-RD. While transcriptional analysis in muscle connective tissue fibroblasts may be preferable given the muscle phenotype, they are not readily available. Patients with COL6-RD also have significant clinical findings in the skin including keloid scarring and hyperkeratosis pilaris making skin fibroblasts a valuable tool for both the diagnosis and study of pathogenies of COL6-RD.[16] We anticipate that key transcriptional pathways regulating both muscle and skin fibroblast phenotypes can be identified with our approach. Differentially expressed genes between COL6-RD subjects (with either DN or null mutations) and controls centered on two themes: an increase in expression of genes in the extracellular matrix and a decrease in expression of genes involved with inflammation and immune response. Matrix-related genes including multiple collagens are upregulated in COL6-RD samples, while multiple integrin and ECM signaling genes are downregulated (Fig 2). Inflammatory regulators including *Il1B*, *IL24* and multiple ELR+ CXC chemokines (most notably *CXCL5* and *Il8*) are downregulated (Fig 3). Since collagen VI is retained intracellularly in fibroblasts from individuals with DN mutations,[6, 16] we hypothesized that the retained collagen VI may induce ER stress; however, genes associated with ER stress were not differentially expressed.

Upregulation of matrix components and downregulation of inflammatory mediators in both DN and null samples compared to controls may be reflected in a dysregulation of normal wound healing processes in absence of normal collagen VI. Wound healing is a complex process including an intial inflammatory response and recruitment of fibroblasts and inflammatory cells, production and deposition of extracellular matrix, and matrix organization and remodeling. This combination of upregulation of matrix components and downregulation of genes controlling matrix remodeling may result in disruption of the balance of matrix production and remodeling and result in overproduction of matrix leading to fibrosis. In this respect collagen VI is thought to be a significant regulator of matrix assembly and composition.[17]

Prior analyses of transcriptional changes in COL6-RD using microarray in both muscle and dermal fibroblast samples both described upregulation of ECM genes, and disruption or ECM remodeling.[18, 19] These authors identified an upregulation of both IGF-1 and follistatin genes in muscle samples from COL6-RD subjects. In our study, *IGFBP2* (Insulin-Like Growth Factor Binding Protein 2) is one of the most significantly upregulated genes with log_2_ fold change of +4.0 in control vs. DN and +3.3 in control vs. null samples. *IGFBP2* is thought to be an important regulator of wound healing by its effect promoting proliferation and differentiation of fibroblasts and myoblasts.[20, 21] In agreement with previous findings that *FST* (follistatin) is upregulated in muscle biopsy samples form COL6-RD individuals, in our study, *FST* in dermal fibroblasts is significantly upregulated (log_2_ fold change +1.4 in DN and +1.2 in Null). *FST* is of particular interest since follistatin is a natural myostatin antagonist and has been proposed as a target for treatment of muscular dystrophy by promotion of muscle growth and decreasing fibrosis.[22, 23] Upregulation of *FST* in COL6-RD subjects may reflect an active response to progressing fibrosis.

COL6-RD are unusual in that inheritance can be either dominant or recessive. In most cases, dominant mutants produce an abnormal, dysfunctional matrix, while recessive mutations result in absent collagen VI in the matrix. While similar in many respects, DN and null subjects have key differences in DE genes compared to controls. *THBS4* (thrombospondin 4, log_2_ fold change +3.1) and *TNXB* (tenascin XB, log_2_ fold change +1.5) were markedly upregulated in DN vs. control samples but not null vs. control samples, perhaps reflecting the role of abnormal collagen VI in the ECM of DN fibroblasts which is not present in null fibroblasts. Thrombospondins, including *THBS4* are secreted proteins that are embedded in the ECM and play a role in angiogenesis and wound healing, and may play a role in regulation of the composition of the ECM in skeletal muscle.[24] Overexpression of mouse *Thbs4* induces an ATF6-dependent endoplasmic reticulum stress response after cardiac injury that induces one branch of the UPR gene expression program, including increasing the protein levels of BiP (Grp78), Sdf2l1, Creld2, Calr, Armet, Hyou1, Mthfd2, and PDI.[25] In our study, only *SDF2L1* from this pathway was differentially expressed in the DN vs. control comparison, although the decreased expression level in DN fibroblast suggests this was not an ER stress-induced response. It has also been suggested that Thbs4 may facilitate the trafficking of ECM proteins through the vesicular secretory pathway.[26] Therefore, *THBS4* upregulation in both DN and null fibroblast may also reflect a change in the regulation of intracellular trafficking in response to unbalanced collagen VI levels in the secretory pathway. *TNXB* is a glycoprotein in the ECM with significant role in matrix maturation and wound healing. Biallelic mutations in *TNXB* are associated with classical-like Ehlers-Danlos syndrome, and share many symptoms with COL6-RD.[27–30] *COMP* (cartilage oligomeric matrix protein) is another thrombospondin family gene (thrombospondin 5) with a role in bridging ECM structures and fibrosis.[31] *COMP* is one of the most significant DE genes with log_2_ fold change of +1.7 in DN vs. control fibroblasts but no significant difference between null and control fibroblasts.

A comparison of transcriptional profiles between DN and null samples reveals a striking down-regulation of genes involved in cell division and proliferation pathways in null samples. While the upregulation of genes promoting production of ECM and downregulation of regulators of inflammation and tissue remodeling (wound healing) may not be surprising in a disorder such as COL6-RD, the down-regulation of cell division and proliferation pathways is less intuitive, and may reflect a role for collagen VI in regulating cell proliferation. In that regard, soluble collagen VI has been shown to stimulate cell proliferation and DNA synthesis in a variety of cell-culture systems.[32] This effect is thought to be regulated through promotion of G1 to S phase by cyclins A, B, and D1.[33] In our study, cyclins B1, B2 and D1 were all significantly downregulated in null samples which lack collagen VI in the ECM compared to DN (log_2_ fold change for *CCNB1*–0.61, *CCNB2*–0.68, and for *CCND1*–0.80). The difference in transcription of genes associated with proliferation and cell division between DN and null may reflect the absence of collagen VI in the matrix of null samples. Consistent with this idea, individuals with collagen VI null mutations lack a soluble cleavage product of the C5 domain of the collagen VI α3(VI) chain, referred to as endotrophin, which has been identified as a soluble regulator of tumor progression in several cancers and a mediator of metabolic regulation.[34–36]

Collagen VI is an important component of the extracellular matrix of many tissues including muscle, skin, tendon, cartilage, and adipose tissues, and has an important role in maintaining structural stability of tissues by anchoring the basement membrane to the extracellular matrix.[37–39] It is not yet clear how a defect in collagen VI, which is produced primarily by muscle connective tissue fibroblasts results in a defect in the muscle.[4, 6, 7] It has been suggested that the simultaneous presence of myofiber atrophy and regeneration seen in collagen VI myopathy may represent a failure of myogenesis and repair mechanisms that are dependent on interactions between the MCT fibroblasts and the satellite cell.[40, 41] Here we have identified differentially expressed genes in fibroblast samples form COL6-RD subjects that reflect the importance of pathways controlling inflammation and production of ECM as well as highlight the importance of collagen VI in regulating proliferation. Our results suggest that a defect in the balance of ECM synthesis and breakdown in COL6-RD may result in increased matrix deposition, leading to early fibrosis.

## Supporting information

S1 Fig Immunofluorescent staining of cultured fibroblasts from a COL6-RD individual (subject 15) with a homozygous null mutation in COL6A3 (p.S18*). Staining for collagen VI in cultured fibroblasts from subject 15 shows absence of collagen VI in the ECM of patient fibroblasts compared to control. Collagen VI staining in the presence of TritonX-100 to permeablize the cell membrane demonstrates absence of intracellular retention of collagen VI that is typical samples with dominant negative mutations. Immunofluorescence analysis of cultured fibroblasts was performed as described by Lampe et al., *Hum Mutat*. 2008; 29:809–822. Fibroblasts were grown to 80% confluence and then treated with L-ascorbic acid (50ng/μl) for 5 days and then fixed in 4% parformaldehyde and blocked with 10% fetal bovine serum albumin with or without 0.1% TritonX-100. Staining for collagen VI was performed using anti-collagen VI monoclonal antibody MAB3303 (Chemicon, Temecula, CA) 1:2,500 dilution, and Alexa Fluor 488-conjugated goat anti-mouse immunoglobulin (Molecular Probes, Eugene, OR) 1:500 dilution. Images were obtained using a Nikon Eclipse Ti microscope.
(EPS)

S2 Fig Multidimensional scaling analysis for differentially expressed genes in COL6-RD and control fibroblasts. COL6-RD fibroblast samples with dominant negative mutations (n = 8), null mutations (n = 5), and controls (n = 6) showing clustering by mutation class based on gene expression levels.
(EPS)

S1 File Significant Differentially Expressed Genes in control and COL6-RD fibroblasts with dominant negative or null mutations. (XLSX)

S2 File Significant DE genes within enriched Gene Ontology categories. (XLSX)

S1 Table KEGG categories with significant enrichment of differentially expressed (DE) genes. (DOCX)
