# Supplementary material for: Transcriptome profiling identifies regulators of pathogenesis in collagen VI related muscular dystrophy
Source: PLoS One. 2017 Dec 15;12(12):e0189664. doi: 10.1371/journal.pone.0189664 (PMC5731705; doi:10.1371/journal.pone.0189664)
Supplement: S1 Table — (DOCX) [file pone.0189664.s005.docx]

**Supplemental Table 1. KEGG categories with significant enrichment of differentially expressed (DE) genes.**

| **Enriched KEGG Category** | **KEGG ID** | **FDR (BH)** | **DE Genes in Category** | **All Genes in Category** |
| --- | --- | --- | --- | --- |
| **Control vs. Dominant Negative** |  |  |  |  |
| Cytokine-cytokine receptor interaction | hsa04060 | 1.45E-03 | 18 | 74 |
| Rheumatoid arthritis | hsa05323 | 1.29E-02 | 11 | 40 |
| Hematopoietic cell lineage | hsa04640 | 5.33E-02 | 8 | 27 |
| Lysosome | hsa04142 | 6.00E-02 | 17 | 98 |
| ECM-receptor interaction | hsa04512 | 6.81E-02 | 11 | 50 |
| Axon guidance | hsa04360 | 8.57E-02 | 13 | 69 |
| Malaria | hsa05144 | 9.73E-02 | 6 | 20 |
|  |  |  |  |  |
| **Control vs. Null** |  |  |  |  |
| Cytokine-cytokine receptor interaction | hsa04060 | 2.44E-04 | 14 | 77 |
| Rheumatoid arthritis | hsa05323 | 1.28E-03 | 9 | 41 |
| Wnt signaling pathway | hsa04310 | 3.82E-03 | 14 | 95 |
| Basal cell carcinoma | hsa05217 | 5.76E-03 | 7 | 26 |
| Pathways in cancer | hsa05200 | 8.24E-03 | 21 | 207 |
| Cell cycle | hsa04110 | 4.27E-02 | 12 | 108 |
| Melanogenesis | hsa04916 | 4.27E-02 | 8 | 51 |
| Amoebiasis | hsa05146 | 4.27E-02 | 8 | 51 |
| Protein digestion and absorption | hsa04974 | 5.97E-02 | 6 | 30 |
| Hedgehog signaling pathway | hsa04340 | 6.52E-02 | 5 | 23 |
| Chagas disease (American trypanosomiasis) | hsa05142 | 7.00E-02 | 8 | 63 |
| Retinol metabolism | hsa00830 | 7.81E-02 | 3 | 9 |
| Hematopoietic cell lineage | hsa04640 | 8.02E-02 | 5 | 27 |
| Small cell lung cancer | hsa05222 | 8.74E-02 | 8 | 62 |
| Axon guidance | hsa04360 | 9.79E-02 | 9 | 76 |
| Hypertrophic cardiomyopathy (HCM) | hsa05410 | 9.96E-02 | 6 | 39 |
|  |  |  |  |  |
| **Dominant Negative vs. Null** |  |  |  |  |
| DNA replication | hsa03030 | 3.04E-12 | 22 | 34 |
| Mismatch repair | hsa03430 | 9.14E-04 | 10 | 20 |
| Cell cycle | hsa04110 | 1.45E-03 | 27 | 110 |
| Pyrimidine metabolism | hsa00240 | 4.54E-03 | 20 | 79 |
| Malaria | hsa05144 | 2.79E-02 | 8 | 20 |
| Nucleotide excision repair - | hsa03420 | 4.00E-02 | 11 | 39 |
| ECM-receptor interaction | hsa04512 | 4.00E-02 | 14 | 50 |
| Rheumatoid arthritis | hsa05323 | 4.00E-02 | 11 | 40 |
| Base excision repair | hsa03410 | 6.84E-02 | 9 | 31 |
| Purine metabolism | hsa00230 | 7.34E-02 | 21 | 109 |
| Jak-STAT signaling pathway | hsa04630 | 9.34E-02 | 15 | 67 |
